# High prevalence of mixed infections in global onychomycosis

**DOI:** 10.1371/journal.pone.0239648

**Published:** 2020-09-29

**Authors:** Aditya K. Gupta, Valeria B. A. Taborda, Paulo R. O. Taborda, Avner Shemer, Richard C. Summerbell, Kerry-Ann Nakrieko

**Affiliations:** 1 Department of Medicine, the University of Toronto, Toronto, Ontario, Canada; 2 Mycology Section, Mediprobe Research Inc., London, Ontario, Canada; 3 Cosmetic Dermatology and Laser Center (Delcentro), Bauru, São Paulo, Brazil; 4 Sackler School of Medicine, Chaim Sheba Medical Center, the Tel-Aviv University, Tel-Hashomer, Israel; 5 Sporometrics, Toronto, Ontario, Canada; 6 Dalla Lana School of Public Health, University of Toronto, Toronto, Ontario, Canada; University of California Riverside, UNITED STATES

## Abstract

Onychomycosis is estimated at a prevalence of 10% worldwide with the infecting organism most commonly *Trichophyton rubrum* (*T*. *rubrum*). Traditional culture identification of causative organisms has inherent risks of overestimating dermatophytes, like *T*. *rubrum*, by inhibiting the growth of possible nondermatophyte mould (NDM) environmental contaminants which could be causative agents. Recently, molecular methods have revealed that a proportion of onychomycosis cases in North America may be caused by mixed infections of *T*. *rubrum* as an agent co-infecting with one or more NDM. Determining the global burden of mixed infections is a necessary step to evaluating the best therapies for this difficult-to-treat disease. To determine the prevalence of mixed infections in a global population, nail samples from onychomycosis patients in Brazil, Canada, and Israel (n = 216) were analyzed by molecular methods for the presence of dermatophytes and five NDMs. If an NDM was detected, repeat sampling was performed to confirm the NDM. *T*. *rubrum* was detected in 98% (211/216) of infections with 39% mixed (84/216). The infection type was more likely to be mixed in samples from Brazil, but more likely to be a dermatophyte in samples from Canada and Israel (Χ^2^ = 16.92, df = 2, *P*<0.001). The most common cause of onychomycosis was *T*. *rubrum*. In all countries (Brazil, Canada and Israel combined) the prevalence of dermatophyte (Χ^2^ = 211.15, df = 3, *P*<0.001) and mixed (dermatophyte and NDM; Χ^2^ = 166.38, df = 3, *P*<0.001) infection increased with patient age. Our data suggest that mixed infection onychomycosis is more prevalent than previously reported with the aging population being at increased risk for mixed infections.

## Introduction

It is estimated that onychomycosis is at a prevalence of 10% worldwide [1] and that the most common causative agent is *Trichophyton rubrum* (*T*. *rubrum*) [2]. In a patient who has abnormal appearing nails suggestive of onychomycosis it is important to obtain the correct identity of the infective fungal organisms so that appropriate management can be instituted. In addition to a dermatophyte infection it is especially important to diagnose a nondermatophyte mould (NDM) infection or mixed infection (dermatophyte and NDM) so that the most appropriate antifungal agent is selected. Thus, the means of identification of etiological agents of onychomycosis is paramount.

Traditional culture-based identification techniques have been compared to molecular identification methods; false negative cultures have been shown to account for most disagreements between methods [3]. Factors such as the mosaic of actively growing and moribund patches of fungal growth contribute to high false negative culture rates of up to 50% as judged by comparison with direct microscopic potassium hydroxide examination or molecular identification [3, 4]. Obtaining a “pure” culture of a dermatophyte with the use of selective antifungals, such as cycloheximide, impedes the growth of other possible *bona fide* etiological agents, like many of the NDM(s). The unique challenges of isolating causative NDM(s) may cause underestimation of their clinical prevalence; for example, aggressive outgrowth by one co-infecting microorganism in culture may also mask other etiological agents. Therefore, repeat sampling with repeat isolation of NDM(s) from culture will not only increase the likelihood that potentially masked organisms are identified, but will also increase the likelihood that the organisms cultured are causative agents and not environmental contaminants [5]. The high sensitivity of molecular identification techniques offers a means of ensuring all causative agents are identified, although the spate of identifications obtained may include environmental contaminants that can only be considered clinically significant with a full mycological examination including direct microscopy and repeat sampling. The present study is the first to survey the causal agents of onychomycosis on a global scale with the use of molecular identification techniques.

## Materials and methods

The study was approved by Institutional Review Board Services (Aurora, ON, Canada). Written consent was obtained.

### Clinical samples and reference organisms

Scrapings of the toenail plate (1–5 mm^2^) were obtained from patients with clinical onychomycosis. A total of 534 samples were analyzed from 216 subjects from Brazil (n = 54), Canada (n = 125) and Israel (n = 37). If a dermatophyte was identified then a repeat sample was not required; however, if an NDM was identified then one or more repeat samples were obtained. The following organisms were used as reference strains: *Acremonium spinosum* (American Type Culture Collection (ATCC) 9471, Manassas, VA, USA), *Aspergillus fumigatus* (ATCC KM8001), *Fusarium oxysporum* (ATCC 26225), *Microsporum audouinii* (Clinical isolate, Mediprobe Research, Inc., London, ON, Canada), *Microsporum canis* (ATCC 32507), *Scopulariopsis brevicaulis* (ATCC 52175), *Neoscytalidium dimidiatum* (ATCC 46921), *Trichophyton mentagrophytes* (ATCC MYA-4439), *Trichophyton rubrum* (ATCC MYA-4438), and *Trichophyton tonsurans* (ATCC 10217).

### DNA isolation and molecular determination of infecting microorganisms

S1 Table summarizes the molecular techniques used for the identification of dermatophyte and NDMs: genes targeted, primers, amplicon sizes and restriction enzymes. Fungal DNA was extracted from toenail samples (1–5 mg) or each reference microorganism (5–10 mg) by adding 50 μL QuickExtract DNA 1.0 extraction solution (Epicentre, Madison, WI, USA) per 5 mg, incubating at 65°C for 45 min, boiling for 2 min, and cooling to room temperature. Reference and sample DNA (7.0 μL) were used for all initial PCR analyses, and 1.0 μL of PCR reaction mixture after thermocycling was used for each nested PCR.

Negative PCR results were repeated once: 3.0 μL of DNA was used for repeated PCRs and 2.0 μL of PCR reaction mixture after thermocycling was used for repeated nested PCRs. Dermatophytes [6], *Acremonium spp*. [4] and *Neoscytalidium spp*. [7] were identified by restriction fragment length polymorphisms (RFLPs) of the PCR products. *Aspergillus spp*., *F*. *oxysporum*, and *S*. *brevicaulis* were identified and *T*. *rubrum* confirmed by nested PCR [8].

### Statistical analyses

Analyses were performed on data collected from the 216 subjects between the years 2009 to 2020. Data were coded categorically for infecting organism(s), country, age, and infection type (mixed or dermatophyte(s) alone). For repeat samples from the same subject, the age was determined as the age of the subject at the first visit. The results for identified infecting organisms in repeat samples from the same subject were summarized into a single entry as follows: dermatophyte species were considered positive if positive in one sample (dermatophyte negative if negative in all samples), and NDM species were considered positive if positive in two or more repeat samples (NDM negative if positive in one sample and negative in repeat samples). The results for identified infecting organisms in single samples were as follows: a dermatophyte positive result concomitant with NDM negative results were included in the data set as dermatophyte positive (as a repeat sample with an NDM positive result would not change the infection status). If a dermatophyte was identified in the same sample as a positively identified NDM and repeat sampling was not possible, then the sample was not included in the data. If an NDM was identified and that sample was negative for a dermatophyte, and repeat sampling was not possible, then the sample was not included in the data.

Coded data were analyzed in two ways: 1) using IBM SPSS Statistics for Windows, version 20.0 (IBM Corp, Armonk, NY, USA) to comparatively measure frequencies of categories within groups with the Pearson’s *chi*-square (*Χ*^*2*^) test and 2) using RStudio, version 1.1.463 (RStudio Team, 2015: Integrated Development for R, Boston, MA, USA) to determine whether there was a relationship between age and frequency of dermatophyte and mixed infections with the *chi*-square goodness-of-fit test. To determine the significance of age and infection status, age was categorized into four groups: (1) below 20 years, (2) between 20 and 30 years, (3) between greater than 30 and 40 years and (4) above 40 years. We carried out this test on individual countries and on the entire sample that constituted the three countries. *P*<0.05 was deemed significant.

## Results

The onychomycosis patients (N = 216; n = 90 female, n = 126 male) were 12 to 90 years old, with a mean age of 56 years (16SD). The most prominent infection type globally was dermatophyte infections (Table 1). *T*. *rubrum* was detected in the majority of infections at 98% (211/216), either as a single organism (60%, 129/216) or as a co-infecting organism (38%, 82/216; Table 2). In Israel and Brazil, *T*. *rubrum* was present as an etiologic agent in all infections seen, serving as the sole agent in 78% (29/37) of cases in Israel and in 39% (21/54) of cases in Brazil. The remaining cases had co-infecting agents (Table 2). In Canada, *T*. *rubrum* infections accounted for 96% (120/125) of infections, with this organism serving as sole agent in 63% (79/125) of cases and as a co-infecting organism in 33% (41/125; Table 2). A member of the *T*. *mentagrophytes* complex was seen as a dermatophytic etiologic agent in a small number of Canadian cases (14 cases total; sole agent in one case; Table 2).

**Table 1:** Infection type in global onychomycosis by country.

Infection Type	Brazil	Canada	Israel	Total
Dermatophyte(s)	39% (21/54)	66% (82/125)	78% (29/37)	61% (132/216)
Mixed*	61% (33/54)	34% (43/125)	22% (8/37)	39% (84/216)

*Mixed, infection of both dermatophyte(s) and nondermatophyte mould(s).

**Table 2:** Etiological agent(s) of global onychomycosis by country.

Etiological Agent(s)	Brazil	Canada	Israel	Total
*T*.*R*.	39% (21/54)	63% (79/125)	78% (29/37)	60% (129/216)
*T*.*R*. + Asp.	33% (18/54)	15% (19/125)	14% (5/37)	19% (42/216)
*T*.*R*. + *F*.*O*.	2% (1/54)	4% (5/125)	0	3% (6/216)
*T*.*R*. + *S*.*B*.	2% (1/54)	4% (5/125)	0	3% (6/216)
*T*.*R*. + *Asp*. + *S*.*B*.	9% (5/54)	0	3% (1/37)	3% (6/216)
*T*.*R*. + *Asp*. + *F*.*O*.	4% (2/54)	2% (2/125)	3% (1/37)	2% (5/216)
*T*.*R*. + *T*.*M*. + *Asp*.	0	3% (4/125)	0	2% (4/216)
*T*.*M*.+ *F*.*O*.	0	2% (3/125)	0	1% (3/216)
*T*.*R*. + *T*.*M*.	0	2% (2/125)	0	<1% (2/216)
*T*.*R*. + *Neo*. + *S*.*B*.	4% (2/54)	0	0	<1% (2/216)
*T*.*R*. + *Acre*. + *Asp*. + *F*.*O*.	4% (2/54)	0	0	<1% (2/216)
*T*.*R*. + *Acre*. + *Asp*. + *S*.*B*.	4% (2/54)	0	0	<1% (2/216)
*T*.*M*.	0	1% (1/125)	0	<0.5% (1/216)
*T*.*M*. + *S*.*B*.	0	1% (1/125)	0	<0.5% (1/216)
*T*.*R*. + *Neo*.	0	1% (1/125)	0	<0.5% (1/216)
*T*.*R*. + *T*.*M*. + *F*.*O*.	0	1% (1/125)	0	<0.5% (1/216)
*T*.*R*. + *Acre*. + *Asp*.	0	0	3% (1/37)	<0.5% (1/216)
*T*.*R*. + *T*.*M*. + *Acre*. + *Asp*.	0	1% (1/125)	0	<0.5% (1/216)
*T*.*R*. + *T*.*M*. + *Asp*. + *S*.*B*.	0	1% (1/125)	0	<0.5% (1/216)

Acre., Acremonium species; Asp., Aspergillus species; F.O., Fusarium oxysporum; Neo., Neoscytalidium species

T.M., Trichophyton mentagrophytes; T.R., Trichophyton rubrum; S.B., Scopulariopsis brevicaulis.

Mixed infections, where NDM(s) were detected as a co-infecting organism(s) in addition to a dermatophyte(s), were detected in 39% (84/216) of global infections (Table 1). By country, the prevalence of mixed infections was as follows: 61% (33/54) in Brazil, 34% (43/125) in Canada and 22% (8/37) in Israel (Table 1). The infection type was analyzed by country with Pearson’s *chi*-square test (Χ^2^ = 16.92, df = 2, *P*<0.001) and resulted as significant, where it is more likely to have a mixed result detected in samples from Brazil, but more likely a dermatophyte result in samples from Canada and Israel (Table 1).

In all three countries combined, as well as individually, the frequency of dermatophyte infection increased significantly with age (Table 3). Similarly, the frequency of mixed infections increased significantly with age in all countries combined (Brazil, Canada and Israel) and in Brazil and Canada when considered separately (Table 3). The relationship was non-significant for Israel alone (*P* = 0.236), although our result for Israel supports the trend in which frequency of mixed infections increases with age (Table 3). The non-significant finding for Israel could be an artifact of the small number of mixed infections obtained.

**Table 3:** Onychomycosis infection type by country and age.

	Counts of dermatophyte infection				Counts of mixed infection			
Age	Total	Brazil	Canada	Israel	Total	Brazil	Canada	Israel^a^
(years)	(n = 132)	(n = 21)	(n = 82)	(n = 29)	(n = 84)	(n = 33)	(n = 43)	(n = 8)
**<20**	3 (2%)	0	3 (4%)	0	0	0	0	0
**20–30**	11 (8%)	4 (19%)	5 (6%)	2 (7%)	5 (6%)	5 (15%)	0	0
**>30–40**	13 (10%)	6 (29%)	6 (7%)	1 (3%)	7 (8%)	4 (12%)	2 (5%)	1 (12%)
**>40**	105 (80%)	11 (52%)	68 (83%)	26 (90%)	72 (86%)	24 (73%)	41(95%)	7 (88%)
	*P*<0.001	*P*<0.01	*P*<0.001	*P*<0.001	*P*<0.001	*P*<0.001	*P*<0.001	*P* = 0.236
	df = 3	df = 3	df = 3	df = 3	df = 3	df = 3	df = 3	
	*Χ*^2^ = 211.15	*Χ*^2^ = 11.95	*Χ*^2^ = 146.98	*Χ*^2^ = 64.93	*Χ*^2^ = 166.38	*Χ*^2^ = 41.79	*Χ*^2^ = 113.74	

Note: the frequency of individuals with dermatophyte and mixed infections were compared across four age groups using the *chi*-square goodness of fit test.

^a^Given the small sample size (i.e., n = 8), and therefore expected cell counts of below 5, we used Fischer’s exact test—where we used two age groups (i.e., above 40 years and 40 or below).

All NDM infections were mixed (detected with a co-infecting dermatophyte(s); Tables 1 and 2). The five NDMs that were identified were *Aspergillus spp*.at 30% (64/216), *S*. *brevicaulis* at 8% (18/216), *F*. *oxysporum* at 8% (17/216), *Acremonium spp*. at 3% (17/216), and *Neoscytalidium spp*. at <1% (3/216; Table 2). There were no *Neoscytalidium spp*. identified in samples collected from Israel. The majority of *Acremonium spp*. were identified in individuals from Brazil (4/6; Table 2).

## Discussion

The knowledge of infecting agents of onychomycosis is a necessary precursor to treatment. *T*. *rubrum* was the most common etiological agent of onychomycosis detected in our global study at 98% (211/216) which corroborates all previous molecular-based investigations. This fungus caused 86% (38/44) of infections in a sample taken in North America [9], 76% (199/261) of a European sample [10] and 74.5% (35/50) to 77% (382/496) of a Japanese sample [8, 11]. The high prevalence of NDMs coexisting with *T*. *rubrum* and other dermatophytes is a highlight of the present survey: mixed infections were detected in 39% (84/216) of onychomycosis patients in this global collaboration.

The proportion of mixed infections in this study was 61% in Brazil, 34% in Canada and 22% in Israel. Although the proportions from Canada and Israel are within a 10% similarity of those obtained in molecular studies in North America (41%) [9] and Japan 21% [8], the high prevalence of mixed infections in Brazil is noteworthy in this survey. All subjects with an NDM reported in our study were confirmed with high confidence by repeat sampling in at least one additional positive sample. With the true counts of NDMs in mixed infections having been reasonably estimated, we conclude with confidence that an onychomycosis is most likely to consist of a mixed infection in Brazil, but most likely to be a pure dermatophyte infection in Canada and Israel (Χ^2^ = 16.92, df = 2, *P*<0.001).

The most commonly detected NDM group in this global population was *Aspergillus spp*., which coincides with findings from Japan [8] and India [12]. It is likely that geographical location accounts for the variability among NDM prevalence, as the prevalence of *F*. *oxysporum* (9%) and *S*. *brevicaulis* (6%) in Canada in this study are within 11% and 5% of the counts previously obtained for these NDMs in a subpopulation of 44 North American patients with onychomycosis [9]. These patients yielded *F*. *oxysporum* in 20% of cases and *S*. *brevicaulis* in 11% [9].

In the present study, the increase in onychomycosis caused by both dermatophytes alone and mixed agents rose significantly with age (Χ^2^ = 211.15, df = 3, *P*<0.001). We have previously shown that there is an increase in dermatophyte onychomycosis with age [13, 14]. This is the first report to demonstrate that mixed infections also increase significantly with age, perhaps since the factors predisposing to dermatophyte onychomycosis also regulate susceptibility to NDMs.

In the present study, no cases of onychomycosis were due to an NDM alone. Similar results were seen in a survey done in Guatemala, where 0.76% of 4220 cases were attributed solely to an NDM [15]. A small survey done in North America reported no NDM-only infections among 44 patients [9]. Other reports of NDMs alone causing onychomycosis range from 51.6% of 33 cases in Thailand [16], and 21% of 121 cases in another Thai study [17], 35.3% of 150 cases in India [12], 17.0% of 47 cases in Japan [8], 13.6% of 431 cases in Italy [18], and 7.8% of 907 cases in Canada [14]. With the exception of the Japanese study, these reports were based on traditional culture results adhering to recommended NDM isolation guidelines [5, 19].

Measures must be taken by the clinician to ensure that contaminants are distinguished from causative agents before embarking on NDM treatments with oral antifungal agents, which entail risks of adverse events, such as damage to the liver, or drug interactions. It is highly recommended that, if the first test is positive for an NDM, additional patient sampling be undertaken on a minimum of two separate occasions before initiating treatment, and direct microscopy examination be included in the full mycological evaluation, as recommended for confirmation of NDMs as etiological agents [5, 19, 20]. It is noteworthy that terbinafine resistance is rare in dermatophytes [21, 22] and a logical explanation for apparently failed terbinafine treatments of dermatophyte infections could be the presence of a mixed dermatophyte-NDM infection.

Although onychomycosis is considered a superficial infection, many patients have aggravated ongoing conditions or are prone to cure and relapse. It has been proposed that the presence of an NDM may significantly contribute to lack of cure, and to ongoing infection by perpetuating the cure and relapse cycle. It has yet to be determined to what extent the high prevalence of mixed infections as seen in this global study accounts for poor treatment outcomes or extended treatment durations, but the possibility of this causality is clear. Indeed, a recent study examining outcomes of 121 onychomycosis patients in Thailand reported that dermatophyte infections required half the time to achieve complete cure than was needed in mixed infections: average treatment times were 2 and 4 years, respectively. Less than half of the patients with mixed infections (21/47) achieved a complete cure [17].

## Supporting information

S1 Table Summary of molecular methods used for identification of infecting organisms of onychomycosis. (DOCX)

S1 File Full dataset: Identification of infecting organisms of onychomycosis. (XLSX)

## References

[1] Thomas J, Jacobson GA, Narkowicz CK, Peterson GM, Burnet H, Sharpe C. Toenail onychomycosis: an important global disease burden. J Clin Pharm Ther 2010; 35: 497–519. 10.1111/j.1365-2710.2009.01107.x 20831675

[2] Ghannoum M, Isham N. Fungal Nail Infections (Onychomycosis): A Never-Ending Story? PLoS Pathog 2014; 10(6): e1004105 10.1371/journal.ppat.1004105 24901242 PMC4047123

[3] Gupta AK, Nakrieko KA. Onychomycosis infections: Do Polymerase Chain Reaction and Culture Reports Agree? J Am Podiatr Med Assoc 2017; 107: 280–286. 10.7547/15-136 28880601

[4] Bontems O, Hauser PM, Monod M. Evaluation of a polymerase chain reaction-restriction fragment length polymorphism assay for dermatophyte and nondermatophyte identification in onychomycosis. Br J Dermatol 2009; 161: 791–796. 10.1111/j.1365-2133.2009.09291.x 19558597

[5] Summerbell RC, Cooper E, Bunn U, Jamieson F, Gupta AK. Onychomycosis: a critical study of techniques and criteria for confirming the etiological significance of nondermatophytes. Med Mycol 2005; 43: 39–59. 10.1080/13693780410001712043 15712607

[6] Jackson CJ, Barton RC, Evans EG. Species Identification and Strain Differentiation of Dermatophyte Fungi by Analysis of Ribosomal-DNA Intergenic Spacer Regions. J Clin Microbiol 1999; 37: 931–936. 10.1128/JCM.37.4.931-936.1999 10074504 PMC88627

[7] Machouart-Dubach M, Lacroix C, de Chauvin MF, Le Gall I, Giudicelli C, Lorenzo F, et al Rapid Discrimination among Dermatophytes, *Scytalidium* spp., and Other Fungi with a PCR-Restriction Fragment Length Polymorphism Ribotyping Method. J Clin Microbiol 2001; 39: 685–690. 10.1128/JCM.39.2.685-690.2001 11158128 PMC87797

[8] Ebihara M, Makimura K, Sato K, Abe S, Tsuboi R. Molecular detection of dermatophytes and nondermatophytes in onychomycosis by nested polymerase chain reaction based on 28S ribosomal RNA gene sequences. Br J Dermatol 2009; 161: 1038–1044. 10.1111/j.1365-2133.2009.09249.x 19566663

[9] Gupta AK, Nakrieko KA. Molecular Determination of Mixed Infections of Dermatophytes and Nondermatophyte Molds in Individuals with Onychomycosis. 2014; J Am Podiatr Med Assoc 104: 330–336. 10.7547/0003-0538-104.4.330 25076075

[10] Kardjeva V, Summerbell R, Kantardjiev T, Devliotou-Panagiotidou D, Sotiriou E, Gräser Y. Forty-Eight-Hour Diagnosis of Onychomycosis with Subtyping of *Trichophyton rubrum* Strains. J Clin Microbiol 2006; 4: 1419–1427. 10.1128/JCM.44.4.1419-1427.2006 PMC1448676 16597871

[11] Shimoyama H, Satoh K, Makimura K, Sei Y. Epidemiological survey of onychomycosis pathogens in Japan by real-time PCR. Med Mycol 2018; 0: 1–6. 10.1093/mmy/myy096 30380094

[12] Raghavendra KR, Yadav D, Kumar A, Sharma M, Bhuria J, Chand AE. The nondermatophyte molds: Emerging as leading cause of onychomycosis in south-east Rajasthan. Indian Dermatol Online J. 2015; 6(2): 92–97. 10.4103/2229-5178.153010 25821729 PMC4375773

[13] Gupta AK, Gupta G, Jain HC, Lynde CW, Foley KA, Daigle D, et al The prevalence of unsuspected onychomycosis and its causative organisms in a multi-center Canadian sample of 30 000 patients visiting physicians’ offices. J Eur Acad Dermatol Venereol 2016; 30:1567–1572. 10.1111/jdv.13677 27168494

[14] Gupta AK, Jain HC, Lynde C, Macdonald P, Cooper EA, Summerbell RC. Prevalence and epidemiology of onychomycosis in patients visiting physicians’ offices: a multicenter Canadian survey of 15,000 patients. J Am Acad Dermatol 2000; 43: 244–248. 10.1067/mjd.2000.104794 10906646

[15] Martinez-Herrera EO, Arroyo-Camarena S, Tejada-Garcia, Porras-López CF, Arenas R. Onychomycosis due to opportunistic molds. An Bras Dermatol 2015; 90: 334–337. 10.1590/abd1806-4841.20153521 26131862 PMC4516115

[16] Ungpakorn R, Lohaprathan S, Reangchainam S. Prevalence of foot diseases in outpatients attending the Institute of Dermatology, Bangkok, Thailand. Clin Exp Dermatol 2004; 29: 87–90. 10.1111/j.1365-2230.2004.01446.x 14723731

[17] Salakshna N, Bunyaratavej S, Matthapan L, Lertrujiwanit K, Leeyaphan C. A cohort study of risk factors, clinical presentations and outcomes for dermatophyte, non-dermatophyte and mixed toenail infections. J Am Acad Dermatol 2018; 79: 1145–1146. 10.1016/j.jaad.2018.05.041 29860042

[18] Tosti A, Piraccini BM, Lorenzi S. Onychomycosis caused by nondermatophyte molds: Clinical features and response to treatment of 59 cases. J Am Acad Dermatol 2000; 42: 217–224. 10.1016/S0190-9622(00)90129-4 10642676

[19] Gupta AK, Drummond-Main C, Cooper EA, Brintnell W, Piraccini BM, Tosti A. Systematic review of nondermatophyte mold onychomycosis: Diagnosis, clinical types, epidemiology, and treatment. J Am Acad Dermatol 2012; 66: 494–502. 10.1016/j.jaad.2011.02.038 21820203

[20] Shemer A, Davidovici B, Grunwald MH, Trau H, Amichai B. New criteria for the laboratory diagnosis of nondermatophyte moulds in onychomycosis. Br J Dermatol 2009; 160: 37–39. 10.1111/j.1365-2133.2008.08805.x 18764841

[21] Monod M, Mehul B. Recent Findings in Onychomycosis and Their Application for Appropriate Treatment. J Fungi 2019; 5, 20 10.3390/jof5010020 30813287 PMC6463057

[22] Gupta AK, Nakrieko KA. *Trichophyton rubrum* DNA strain switching increases in patients with onychomycosis failing antifungal treatments. Br J Dermatol 172, 74–80. 10.1111/bjd.13165 24903451

