# Supplementary material for: High prevalence of mixed infections in global onychomycosis
Source: PLoS One. 2020 Sep 29;15(9):e0239648. doi: 10.1371/journal.pone.0239648 (PMC7523972; doi:10.1371/journal.pone.0239648)
Supplement: S1 Table — (DOCX) [file pone.0239648.s001.docx]

**S1** **Table.** **Summary of molecular methods used for identification of infecting organisms of onychomycosis.**

| **Organisms Identified** | **Method**  **(Restriction Enzyme)** | **Target** | **Primers** | **Amplicon Size** | **Reference** |
| --- | --- | --- | --- | --- | --- |
| Dermatophytes^a^ | PCR-RFLP  (*BstN*1) | ITS/5.8S rDNA | ITS1: 5’-TCC GTA GGT GAA CCT GCG G-3’  ITS4: 5’-TCC TCC GCT TAT TGA TAT GC-3’ | 590-750 bp | Jackson et al., 1999 |
| *Trichophyton rubrum^b^* | Nested PCR | 28S rDNA | DPUP F1: 5’-AGTAGAGTGATCGAAAGGTT-3’  DPUP R1: 5’-ATTACGCCAGCATCCGAG-3’  TRUB F1: 5’-CGTCGCCCGTGCACTG-3’  TRUB R1: 5’-GAGCGCGTTCCTCAGTCT-3’ | 273-275 bp  137 bp | Ebihara et al., 2009 |
| *Acremonium spp*. | PCR-RFLP  (*Hinf*1, *Rsa*1) | 28S rDNA | LSU1: 5’-GAT AGC GMA CAA GTA GAG TG-3’  LSU2: 5’-GTC CGT GTT TCA AGA CGG G-3’ | 330-332 bp | Bontems et al., 2009 |
| *Aspergillus spp*. | Nested PCR | 28S rRNA | FUP 28SF1: 5’-AAG CAT ATC AAT AAG CGG AGG-3’  FUP 635: 5’-GGT CCG TGT TTC AAG ACG G-3’  ASP F1: 5’-GCA TTC GTG CCG GTG TAC TT-3’  ASP R1: 5’-TTA CGA CCA TTA TGC CAG CG-3’ | 600-650 bp  170 bp | Ebihara et al., 2009 |
| *Fusarium oxysporum* | Nested PCR | 28S rRNA | FUP 28SF1: 5’-AAG CAT ATC AAT AAG CGG AGG-3’  FUP 635: 5’-GGT CCG TGT TTC AAG ACG G-3’  FOXY F1: 5’-ATC TCT GTA AAG TTC CTT CA-3’ FOXY R1: 5’-CCC AGG GTA TTA CAC GGT-3’ | 600-650 bp  335 bp | Ebihara et al., 2009 |
| *Scopulariopsis brevicaulis* | Nested PCR | 28S rRNA | FUP 28SF1: 5’-AAG CAT ATC AAT AAG CGG AGG-3’  FUP 635: 5’-GGT CCG TGT TTC AAG ACG G-3’  SCOP F1: 5’-CGT CGG ATC AAC CGT CGC TT-3’  SCOP R1: 5’-ACG CCA GCA TCC TTG CAT AC-3’ | 600-650 bp  168 bp | Ebihara et al., 2009 |
| *Neoscytalidium spp* | PCR-RFLP (*BamH*1) | 18S rDNA | DH2L: 5’-TGT ACT GGT CCG GCC GGG-3’  DH1R: 5’-CGG CGG TCC TAG AAA CCA AC-3’ | 200 bp | Machouart-Dubach et al., 2001 |
| ^a^Unique patterns: *Epidermophyton floccosum*, *Microsporum audouinii*, *Microsporum canis, Microsporum gypseum*, *Microsporum persicolor*, *Trichophyton mentagrophytes, Trichophyton terrestre*, *Trichophyton verrucosum*, and *Trichophyton violaceum.* Shared patterns: *Trichophyton rubrum* and *Trichophyton soudanese, Trichophyton equinum* and *Trichophyton tonsurans*, *Trichophyton concentricum* and *Trichophyton erinaceid*, *Trichophyton quinkeanum* and *Trichophyton schoenleinii*. ^b^*Trichophyton rubrum* was confirmed as it has a shared RFLP pattern with *Trichophyton soudanese*.^(Jackson et al., 1999)^. bp, base pairs. PCR, polymerase chain reaction; RFLP, restriction fragment length polymorphisms. | | | | | |
